# Risk stratification and subclinical phenotyping of dilated and/or arrhythmogenic cardiomyopathy mutation-positive relatives: CVON eDETECT consortium

**DOI:** 10.1007/s12471-021-01542-1

**Published:** 2021-02-02

**Authors:** R. W. Roudijk, K. Taha, M. Bourfiss, P. Loh, L. van den Heuvel, M. J. Boonstra, F. van Lint, S. M. van der Voorn, A. S. J. M. te Riele, L. P. Bosman, I. Christiaans, T. A. B. van Veen, C. A. Remme, M. P. van den Berg, J. P. van Tintelen, F. W. Asselbergs

**Affiliations:** 1 Netherlands Heart Institute, Utrecht, The Netherlands; 2 Department of Cardiology, Division Heart and Lungs, University Medical Centre Utrecht, Utrecht University, Utrecht, The Netherlands; 3 Department of Clinical Genetics, Amsterdam UMC, Amsterdam, The Netherlands; 4 Department of Genetics, University Medical Centre Utrecht, University of Utrecht, Utrecht, The Netherlands; 5 Department of Genetics, University Medical Centre Groningen, Groningen, The Netherlands; 6 Department of Medical Physiology, University Medical Centre Utrecht, Utrecht, The Netherlands; 7 Department of Clinical and Experimental Cardiology, Amsterdam University Medical Centre, Amsterdam, The Netherlands; 8 Department of Cardiology, University Medical Centre Groningen, Groningen, The Netherlands; 9 Durrer Centre, Amsterdam, The Netherlands; 10 Institute of Cardiovascular Science, Faculty of Population Health Sciences, University College London, London, UK; 11 Health Data Research UK and Institute of Health Informatics, University College London, London, UK

**Keywords:** Pathogenic variants, Cascade screening, Arrhythmogenic cardiomyopathy, Dilated cardiomyopathy, Plakophilin‑2, Phospholamban

## Abstract

In relatives of index patients with dilated cardiomyopathy and arrhythmogenic cardiomyopathy, early detection of disease onset is essential to prevent sudden cardiac death and facilitate early treatment of heart failure. However, the optimal screening interval and combination of diagnostic techniques are unknown. The clinical course of disease in index patients and their relatives is variable due to incomplete and age-dependent penetrance. Several biomarkers, electrocardiographic and imaging (echocardiographic deformation imaging and cardiac magnetic resonance imaging) techniques are promising non-invasive methods for detection of subclinical cardiomyopathy. However, these techniques need optimisation and integration into clinical practice. Furthermore, determining the optimal interval and intensity of cascade screening may require a personalised approach. To address this, the CVON-eDETECT (early detection of disease in cardiomyopathy mutation carriers) consortium aims to integrate electronic health record data from long-term follow-up, diagnostic data sets, tissue and plasma samples in a multidisciplinary biobank environment to provide personalised risk stratification for heart failure and sudden cardiac death. Adequate risk stratification may lead to personalised screening, treatment and optimal timing of implantable cardioverter defibrillator implantation. In this article, we describe non-invasive diagnostic techniques used for detection of subclinical disease in relatives of index patients with dilated cardiomyopathy and arrhythmogenic cardiomyopathy.

## Introduction

In the Netherlands, pathogenic variants in the plakophilin‑2 (*PKP2*) and phospholamban (*PLN*) gene are the most prevalent genetic predispositions for familial dilated cardiomyopathy (DCM) and arrhythmogenic cardiomyopathy (ACM) [1, 2]. Sudden cardiac death (SCD) may be the first manifestation of disease in asymptomatic relatives of index patients with inherited cardiomyopathy [3]. Pathogenic variants are found in 40% of DCM index patients and 67% of ACM index patients [2, 4]. Due to increased awareness and improved genetic analysis techniques, the number of identified relatives carrying a pathogenic variant without overt clinical disease—currently estimated at 300,000 individuals—is increasing. Despite this genetic risk, only 40–60% of relatives will develop cardiomyopathy; this phenomenon is known as incomplete penetrance of disease [5, 6]. This variability is presumably caused by a combination of variants in specific genes, epigenetic regulation and environmental factors. Furthermore, disease development in relatives varies per specific genotype [5, 6]. For example, in carriers of the R14del variant in *PLN*, resulting in super-inhibition of the sarcoplasmic reticulum calcium pump, disease penetrance is high (75–90%) [7]. In carriers of pathogenic PKP2 variants, which may result in dysfunction of cardiac desmosome proteins, disease penetrance is generally lower (40–60%) [1, 4, 5].

### Genetic and clinical cascade screening

Current guidelines recommend clinical evaluation for first-degree relatives every 1–3 years, starting at 10–12 years of age. Recommended evaluation includes 12-lead electrocardiography, Holter monitoring and cardiac imaging [8]. Cascade screening is aimed to identify relatives at high risk of SCD or cardiomyopathy, who could benefit from early pharmacological and lifestyle intervention or device implantation [3]. However, the optimal age to start cascade screening, the optimal screening intervals and the most adequate combination of tests are currently unknown.

### Electrical and structural myocardial remodelling

**Fig. 1:**
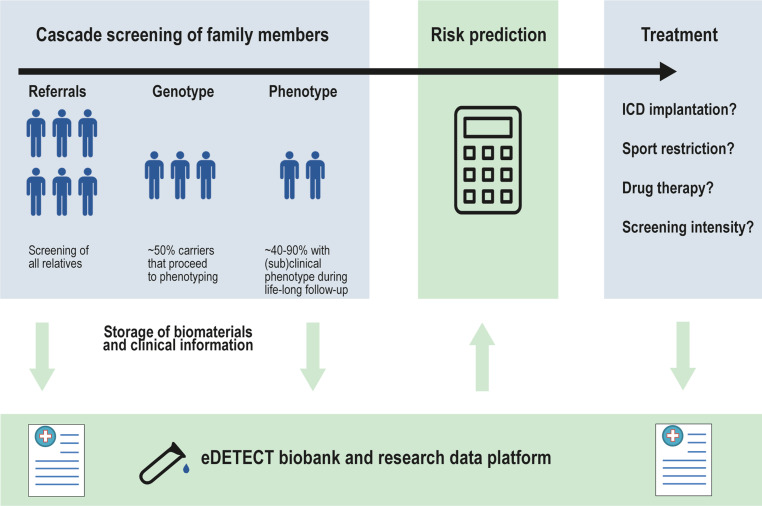
Overview of the integration of cascade screening, individualised risk prediction and individualised treatment into an eDETECT biobank infrastructure. *ICD* implantable cardioverter defibrillator

Inherited cardiomyopathies are characterised by both electrical and structural remodelling of the myocardium. Electrical remodelling promotes heterogeneous conduction delay and ectopic activity, hampers electro-anatomical coupling and enhances arrhythmia susceptibility [9]. Structural remodelling results in contractile impairment and fibrofatty infiltrate formation, resulting in (regional) myocardial dysfunction and ventricular dilatation [9, 10]. Several electrocardiographic methods, imaging modalities and biomarkers are known or have been proposed to assess myocardial remodelling [8]. Ideally, screening methods for asymptomatic relatives are non-invasive, automated, quantitative and user-independent. In this article, we provide an overview of existing and future screening methods for early detection of inherited cardiomyopathies and their integration into the eDETECT consortium and biobank infrastructure (Fig. 1).

## Diagnostic techniques: electrophysiology

Routine 12-lead electrocardiogram (ECG) evaluations are recommended for both index patients and relatives as they have diagnostic and prognostic implications in ACM and DCM [11–13]. In ACM, T‑wave inversions in the precordial leads and non-sustained ventricular tachycardia during Holter monitoring are risk factors for ventricular arrhythmias [8, 14]. In relatives, premature ventricular complexes have been associated with ventricular arrhythmias and disease onset [8, 9, 14]. In DCM, first- or second-degree atrioventricular block, left bundle branch block and prolonged QRS duration are associated with worse prognosis [13]. PR interval prolongation might be the first manifestation of DCM due to *LMNA* or *SCN5A* pathogenic variants [8, 13]. Low-voltage QRS complexes can be observed in carriers of desmosomal and *PLN* pathogenic variants [8, 13]. An important limitation of ECG analysis and Holter monitoring is the short interval of rhythm monitoring opposed to the day-to-day variation of arrhythmias. Wearable monitors might overcome this limitation, thereby providing insight into the risk for SCD and ventricular arrhythmia. Artificial intelligence has proven potential, as it is able to predict atrial fibrillation utilising a 12-lead ECG recording during sinus rhythm [15]. However, further research is required to predict ventricular tachycardia utilising 12-lead ECG recordings.

### Integration of electrocardiography and imaging

Electrocardiographic imaging (ECGi) may be a promising non-invasive technique to identify early electrical remodelling by computing the depolarisation and repolarisation sequences. This technique combines high-resolution ECG recordings from body surface electrodes with cardiac imaging. ECGi has successfully been used to localise arrhythmogenic substrates of atrial fibrillation and ventricular arrhythmias [16]. For example, abnormalities in repolarisation sequence have been successfully identified in patients with Brugada syndrome (steep dispersion of repolarisation in the right ventricular outflow tract), long QT syndrome (steep recovery gradients) and SCD survivors (abnormal recovery of repolarisation) [17].

## Diagnostic techniques: imaging

Non-invasive cardiac imaging modalities such as echocardiography and cardiac magnetic resonance imaging (CMR) are routinely used for screening of relatives and play a key role in diagnosis of cardiomyopathies [8, 9, 12]. However, early signs of disease may go unnoticed by conventional measurements. Advanced techniques such as (echocardiographic or CMR-based) deformation imaging and CMR-based tissue characterisation by T1 mapping might have incremental value in diagnosing subclinical signs of cardiomyopathy.

### Deformation imaging

**Fig. 2:**
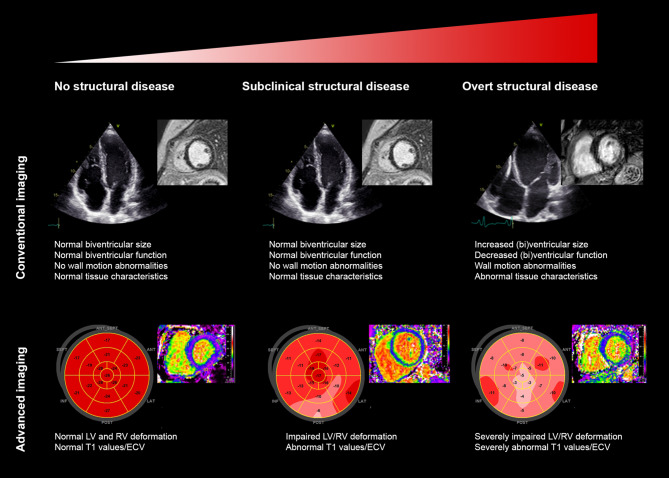
Comparison between conventional and advanced imaging for different stages of disease: no structural disease, subclinical structural disease and overt structural disease. *LV* left ventricle, *RV* right ventricle, *SEPT* septal, *ANT* anterior, *LAT* lateral, *POST* posterior, *INF* inferior, *ECV* extracellular volume

Deformation imaging enables offline quantification of myocardial contractility and has the ability to overcome the limitations of subjective wall motion assessment. This technique is performed either with echocardiography (‘speckle tracking’) or with CMR (‘feature tracking’) and can be used for global and regional assessment of both ventricles [18]. In ACM, deformation imaging was shown to reveal subtle mechanical changes in at-risk relatives [10, 19]. Furthermore, normal deformation patterns in the basal area of the right ventricle were associated with absence of disease progression, while abnormal deformation patterns precede established signs of ACM [20]. Consequently, deformation imaging distinguishes relatives with subclinical disease from those in a truly concealed stage and may therefore be useful in determining screening frequency (Fig. 2).

### Tissue characterisation

T1 mapping enables detection of interstitial myocardial fibrosis in patients with mild disease, in contrast to conventional late gadolinium enhancement, which is able to show only focal fibrosis in patients with advanced structural disease [21]. Indeed, recent studies have shown that abnormal tissue characteristics with T1 mapping are present in the inferior and posterolateral segments in patients with cardiomyopathy as well as carriers of pathogenic variants.

## Diagnostic techniques: biomarkers

### Genetic biomarkers

The specific effects of the pathogenic variant on protein function may serve as a biomarker for disease penetrance; however, this does not predict the timing of disease onset [22]. Prospective genotype-phenotype studies should focus on the clinical applicability of genetic biomarkers. This accounts, for example, for the recently published polygenic risk score, which influences the response to sodium channel blocking drugs in individuals with Brugada syndrome [23].

### Diagnostic biomarkers

The recent discovery of anti-desmoglein 2 (DSG2) autoantibody serum levels may contribute to a promising diagnostic biomarker, since these antibodies were present in all (borderline) ACM patients and absent in control subjects [24]. Although the underlying pathophysiological mechanism and the diagnostic value are unknown, anti-DSG2 autoantibody serum levels are associated with disease severity and arrhythmia incidence (*r* = 0.70 for premature ventricular complex burden).

### Prognostic biomarkers

Several non-specific prognostic biomarkers, such as troponin, B‑type natriuretic peptide (BNP) and *N*-terminal (NT)-pro-hormone BNP (NT-proBNP), are associated with mortality in cardiomyopathies [22]. Importantly, markers of fibrosis (collagen formation and breakdown), markers of inflammation and circulatory microRNAs are expected to be associated with disease severity. However, their applicability as early disease markers is still unknown [25]. In contrast to serum biomarkers, non-invasive analysis of extra-cardiac tissue (such as buccal mucosa) appears to be promising [26]. Through biobanking studies it may be possible to provide a biomarker-based risk profile of individuals. For example, recent advancements in proteomics have combined functional analysis of gut proteins with known heart failure biomarkers (BNP and NTproBNP) to improve their prognostic value [27].

## Integration of diagnostic techniques

The main goal of the CVON-eDETECT consortium is to prevent SCD and to facilitate early treatment of heart failure in relatives by early detection of disease and improved risk stratification. First, we aim to improve participation in genetic counselling and testing in index patients and relatives by improving the provision of information for patients, relatives and health care professionals (https://www.erfelijkehartziekten.nl) [28]. Second, we aim to facilitate prospective genotype-phenotype cohort studies using a biobank structure and to implement advanced diagnostic techniques into clinical practice.

**Fig. 3:**
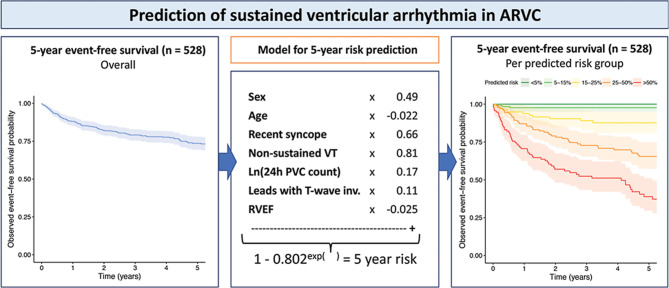
Prediction of sustained ventricular arrhythmia in patients diagnosed with arrhythmogenic right ventricular cardiomyopathy without previous sustained ventricular arrhythmia. *ARVC* arrhythmogenic right ventricular cardiomyopathy,* VT* ventricular tachycardia, *PVC* premature ventricular complex, *T‑wave inv* T-wave inversion, *RVEF* right ventricular ejection fraction. Reproduced with permission [30]

Pre-clinical studies will address underlying disease mechanisms, further facilitating early detection and risk prediction. A biobanking structure with blood and tissue samples combined with imaging and clinical data facilitates this translational approach (data catalogue available at https://www.durrercenter.nl/e-detect/). Long-term clinical follow-up data from the Dutch ACM Registry (median 9.5 (4.6–16.2) years), imaging data and samples from the cardiogenetic biobanks of the Amsterdam University Medical Centre (UMC), UMC Groningen and UMC Utrecht were integrated [4, 29]. To personalise treatment, a risk calculator has been established predicting the risk of ventricular arrhythmias in patients with ACM (https://arvcrisk.com/, Fig. 3; [30]). The CVON-eDETECT consortium aims to create a similar risk calculator to predict SCD and development of cardiomyopathy in relatives. This may aid shared decision-making concerning the implantation of implantable cardioverter defibrillators, sports restriction or drug therapy to reduce progression of heart failure as well as the implementation of a more patient-tailored approach to follow up the intensity and frequency once subclinical disease has been established in relatives.

## Conclusion

Carriers of pathogenic ACM and DCM variants are at risk for SCD and heart failure, and early intervention requires personalised risk stratification. Integration of state-of-the-art imaging and electrophysiological diagnostic techniques into clinical practice is essential to optimise cascade screening. Within CVON-eDETECT, a biobank structure combining clinical phenotypes with genotype, imaging and biomarker information aims to facilitate identification of early disease (bio)markers and risk factors in inherited cardiomyopathy.
